# A new band selection framework for hyperspectral remote sensing image classification

**DOI:** 10.1038/s41598-024-83118-8

**Published:** 2024-12-30

**Authors:** B. L. N. Phaneendra Kumar, Radhesyam Vaddi, Prabukumar Manoharan, L. Agilandeeswari, V. Sangeetha

**Affiliations:** 1 Department of Computer Science and Engineering, Koneru Lakshmaiah Education Foundation, Vaddeswaram, India; 2 Department of Information Technology, Velagapudi Ramakrishna Siddhartha Engineering College (Deemed to be University), Vijayawada, India; 3 School of Computer Science Engineering and Information Systems, Vellore Institute of Technology, Vellore, 632014 India

**Keywords:** Hyperspectral, PCA, Nonlinear, Smoothing filter-weighted least squares, Hyperbolic sigmoid, Spectral, Support vector machine, Environmental sciences, Optics and photonics

## Abstract

Dimensionality Reduction (DR) is an indispensable step to enhance classifier accuracy with data redundancy in hyperspectral images (HSI). This paper proposes a framework for DR that combines band selection (BS) and effective spatial features. The conventional clustering methods for BS typically face hard encounters when we have a less data items matched to the dimensionality of the accompanying feature space. So, to fully mine the effective information, BS is established using dual partitioning and ranking. The bands from the dual partitioning have undergone informative band selection via ranking. The reduced band subset is then given to a hemispherical reflectance-based spatial filter. Then, finally, a Convolutional Neural Network (CNN) is used for effective classification by incorporating three-dimensional convolutions. On a set of three hyperspectral datasets - Indian Pines, Salinas, and KSC, the proposed method was tested with different state-of-the-art techniques. The classification results are compared using quantitative and qualitative measures. The reported overall accuracy is 99.92% on Indian Pines, 99.94% on Salinas, and 97.23% on the KSC dataset. Also, the Mean Spectral Divergence values are 42.4, 63.75, and 41.2 on the three datasets respectively, which signifies the effectiveness of band selection. The results have clearly shown the impact of the band selection proposed and can be utilized for a wide variety of applications.

In recent years, hyperspectral sensing technology has become more popular and is widely used in remote sensing to monitor earth surface information. The hyperspectral sensors have unique acquisition mechanisms and collect abundant information about the earth’s materials covering visible and infrared regions of the electromagnetic spectrum in three dimensions. These results are information on the hyperspectral cube and are used to classify dissimilar materials available in it. The major applications of hyperspectral image classification include crop yield estimation in agriculture^1^; land use land cover mapping^2^, microplastics^3^ and minerals identification^4^, lithological^5^ and defense applications^6^. These can be shown in Fig. 1. Apart from the several applications, the hyperspectral images suffer from different issues such as higher dimensionality, higher correlation, and fewer numbers of labeled samples resulting in less performance in classification. Hence, Dimensionality Reduction (DR) has become inevitable before classification. This paper introduces a novel DR model for selecting the best representative and non-redundant informative bands from the hyperspectral cube. The major categories of DR include BS and feature extraction^7^.

**Fig. 1:**
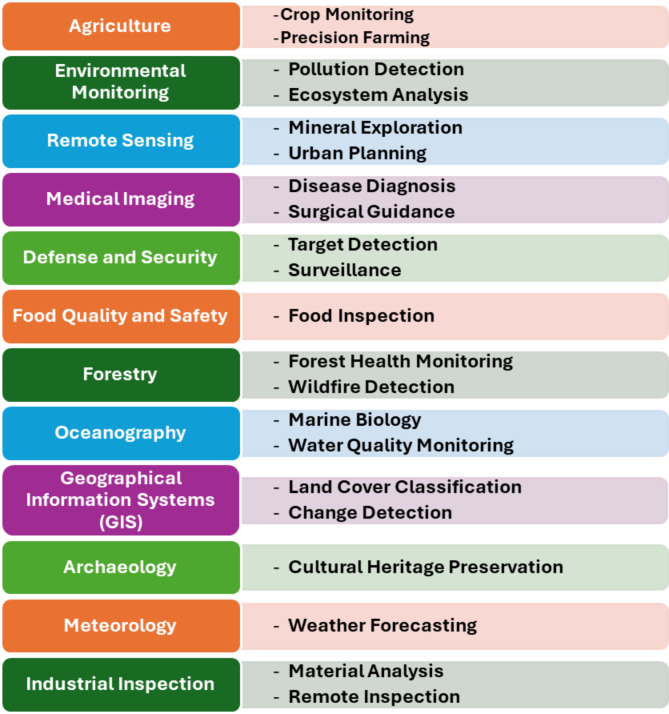
Use cases of hyperspectral image classification.

**Fig. 2:**
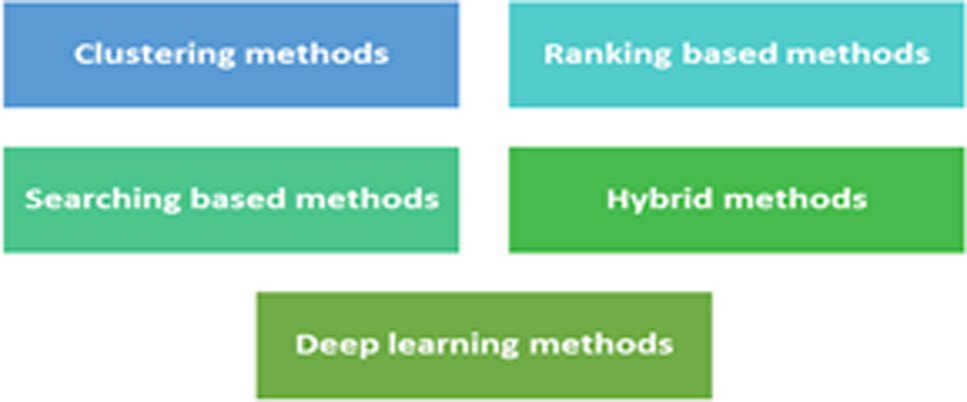
Band selection strategies.

Feature extraction focuses on transforming the original data into a transformed domain as in PCA, PPCA, FA, KPCA, etc., methods^8–16^. A review of various hyperspectral classification methods is given in^17^. The physical meaning of the data is lost in these approaches. Hence, band selection is a good choice for DR for hyperspectral data. The major types of band selection approaches exist in the literature as shown in Fig. 2. The clustering methods initially cluster the bands and then select the representative bands.

In^16^, the authors have introduced a dynamic programming approach for the band selection in which clustering is also taken into consideration. Band selection based on density peak clustering^18^ is utilized for handling non-spherical data in band clustering. Another clustering-based method is proposed which relies on the corr-entropy algorithm for the grouping of spectral bands^19^. A multistage clustering^20^ is proposed. Initial clustering is based on Euclidean distance and a band from each cluster results in a band set (after tuning). Self-tuning algorithm is proposed in^21^. To efficiently capture complex relationships among bands, the kernel function followed by the probabilistic model is used in^22^ to find the relevant informative bands. In^23^, an attention mask is utilized to choose the most representative bands for each pixel, which is then followed by an autoencoder to reconstruct the original image. The ultimate selection of bands is determined through clustering. Another set of algorithms for band selection is ranking-based approaches. Here, the bands are ranked via a pre-defined criterion that evaluates bands. In ranking-based band selection, a pre-defined criterion is considered for evaluating a band. Some of the measures used for prioritization are correlation, signal-to-noise ratio, entropy, neg-entropy, information divergence methods^24,25^. The entropy and divergence-based band selection is highlighted in^26^. So, in^27^, divergence, mutual information, and covariance between the bands are introduced, and the bands are ranked accordingly. A low information-based approach is presented in^25,28^ in which low-correlated bands are extracted by computing the correlation between each band and the entire cube. Here, an impulse response filter is used. A method that computes the absolute difference of mean reflectance of a band and the same computed from a curve fitting is proposed in^29^. Here, ranks are generated based on the different values, and top-rank ones are selected. Two approaches^30,31^ are considered to keep maximum dissimilarity among the bands. A minimum misclassification canonical analysis-based approach is proposed to rank the bands that have a high impact on classification^25^. In recent years, researchers have focused on developing band selection strategies inspired by nature^32^. These are termed nature-inspired heuristic approaches. In^33^, the authors have focused on the comparison of three group intelligence algorithms to focus on the convergence and accuracy for efficient band selection. The measure based on the correlation and cuckoo optimization technique is proposed in^34^, followed by a variation called the modified cuckoo approach so as not to get stuck at the optimal local solution^35^. An optimization technique to overcome the early convergence of wind-driven and modified wind-driven-based band selection is proposed by^36,37^ where a separable band measure is used. A multi-PSO is proposed by^38^ for band selection where entropy and Gaussian filters are used. A band selection based on multi-objective and multi-verse optimization techniques is proposed in^39^. Here, a combination of three fitness (objective) functions to get less redundancy, rich information, and bands to select are utilized for dimensionality reduction. Whale and modified whale optimization approaches for HSI images are proposed in^40,41^. Also, another variation with spectral and spatial information is given in^42^. Based on the Shannon entropy and mutual information measures, a gravitational band search is proposed in^43^. An approach based on Moth-flame optimization is presented in^44^ to effectively select the bands from the hyperspectral images. Recently, multi-objective-based optimization techniques have also been used to select bands from the hyper-spectral images and provide good results^45^. A combination of wind-driven and cuckoo search is implemented in^32^ for informative band selection. To effectively utilize algorithms for band selection, hybrid methods are considered. A ranking and clustering-based method is introduced in^46^ to leverage the inherent structure of HSI data. Here, spectral clustering via self-tuning for band selection is proposed. The parameter adjustment is performed automatically based on the intrinsic structure of data which helps to streamline the band selection. Because of the property that the adjacent bands have a high correlation, an optimal hybrid strategy is proposed in^14^, a combinatorial optimization technique. Minimum noise and entropy measures, a band selection strategy is implemented, and a similarity metric-based strategy is proposed in^47^ followed by a rough set approach for selecting suitable bands, which uses an incremental search strategy, graph-based^11^, machine learning^48^ approaches. Evolutionary with multi-model is proposed in which different subspaces are generated and in each of the subspaces diversified and less correlated bands are obtained^49^. In^50^, an unsupervised clustering in which the entire dataset is clustered initially and iteratively fine-tuned the bands via separating hyperplanes.

Recently, deep learning has been used in HSI. In deep learning, no artificial design features are considered. They automatically learn features or patterns from the hyperspectral data. It includes the architecture of the deep learning model and the learning strategies (supervised or unsupervised)^51,52^. Autoencoder is the first deep learning model implemented by^53^ for hyperspectral images. Spectral-spatial feature extraction-based convolutional network is implemented in^54,55^. The 3D nature of the hyperspectral cube is better utilized for CNN in^56^. A prototype network combined with ResNet is implemented in^57^. Band selection based on attention mechanism^58^, BASS Net^59^, BS Net^60^, and reinforcement learning^61^ are proposed for good classification and crop classification^62^. A review of SVM and CNN is proposed in^63,64^ to get insights into the deep nets. A comparative analysis of different activation functions for hyperspectral images is given by^65^. In recent years, grey wolf optimization (gwo) is used to tune the parameters of deep nets as proposed in^66^. Here, instead of auto-selecting the parameters in CNN, trained the parameters via gwo. The authors have enhanced unsupervised bands election via normalized mutual and variation information to rank the bands^67^. In^68^, proposed an approach to reduce the dimensionality with low reconstruction error. A new approach based on contrastive learning is proposed by^69^ to leverage the transformer mechanism. To enhance the quality in the band selection with similar metrics via improved k-means approach is proposed in^70,71^. Another approach based on spectral & spatial correlation approach is proposed to effectively find the bands^72^.

The major contributions of the paper are as follows:

- Implemented a dual partition strategy for band selection to fully mine the information from the hyperspectral cube (Coarser to finer partition).
  - Initially, the statistical technique, of correlation is utilized for primary partition which partitioned HSI cube based on the correlation coefficient, to maintain the local patterns and neighborhood relationships among each of the primary partitioned band sets, The neighborhood partition algorithm is presented as the second level partition by generating the weight matrix to quantify how each pixel is approximated by neighbor pixels (hence, local structured perseveres) to get different partitions. Then, band prioritization via wavelets from each partition is performed.
- Extracted the intrinsic information from the selected bands using hemispherical reflectance.
- Constructed and trained a three-dimensional convolution neural network that uses three-dimensional convolutions.
- The proposed model is implemented on benchmark datasets and also tested for crop classification.

The rest of the paper is organized as follows: The technicality of the method and the benchmark datasets are provided in Methodology and Datasets respectively. The results analyzed with various parameters are given in Results and analysis followed by the conclusion in Conclusion.

## Methodology

**Fig. 3:**
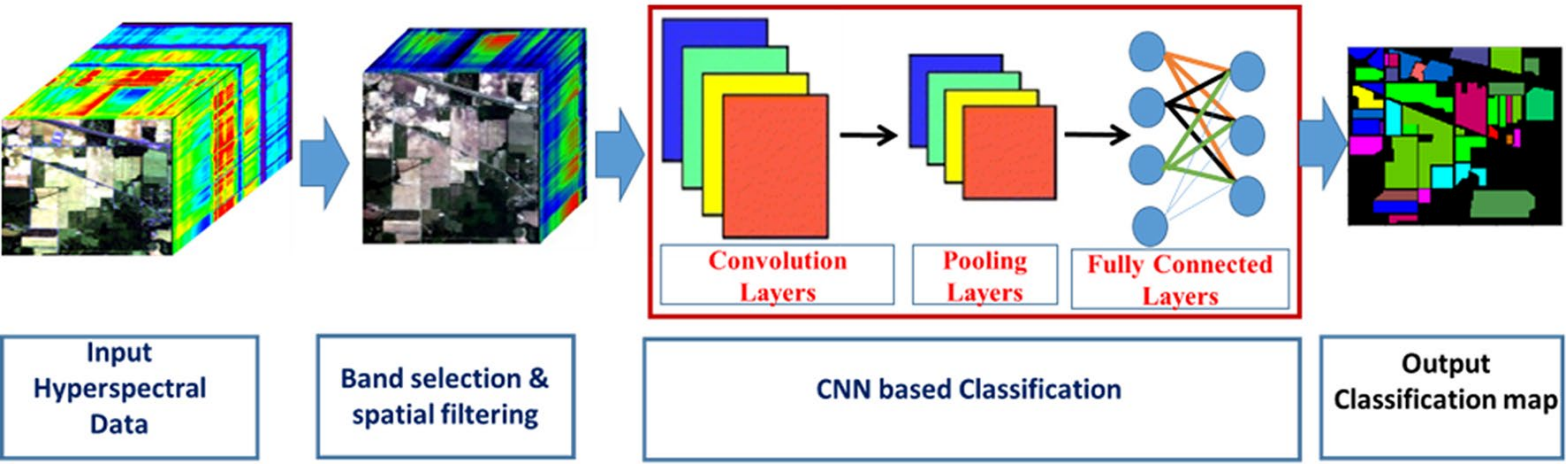
The schematic diagram consists of the major steps of the proposed method.

**Fig. 4:**
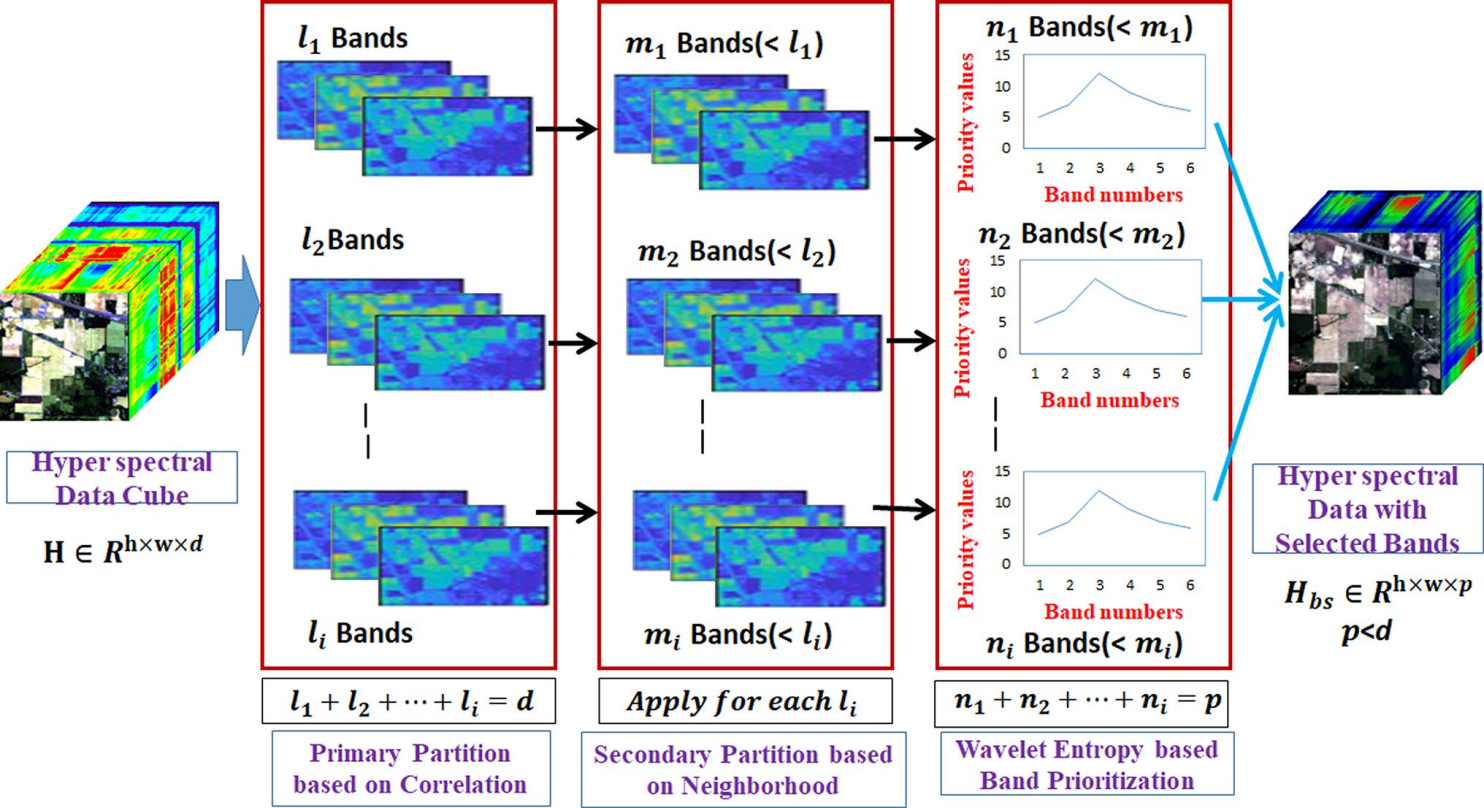
Dual partitioning and priority based band selection.

The methodology of the proposed system is shown in Fig. 3 and consists of major steps followed. The dual Partitioning and Priority-based band selection is shown in Fig. 4. The brief description of the proposed methodology introduced in this paper is outlined hereunder:

Due to the high dimensionality of the HSI cube and the selection of non-redundant and effective features, this paper employs a dual-partitioning strategy with band prioritization. The dual partitioning strategy effectively selected the best informative bands for further classification. Initially, the statistical technique, of correlation is utilized for the primary partitioning HSI cube with d bands into i partitions based on the correlation coefficient. To maintain the local patterns and neighborhood relationships among each of the primary partitioned ‘i’ band sets, the neighborhood partition algorithm is presented as the second level partition by generating the weight matrix to quantify how each pixel is approximated by neighbor pixels (hence, local structured perseveres) to get different partitions. Now, band prioritization via wavelets from each partition is performed to find the regions in the bands with significant and reducing redundant variations. This generates a band set with prioritized bands. Then, a Bi-hemispherical reflectance recovery approach to find the intrinsic spatial features. The generated band set is then trained with convolution neural networks for classification.

### Dual partitioning and band prioritization

Let the data is denoted as $\:\text{H}=\left[{\text{X}}_{1},{\:\text{X}}_{2},\dots\:.{\text{X}}_{\text{d}}\right]\in\:{\text{R}}^{\text{h}\times\:\text{w}\times\:\text{d}}$ with d bands, width ‘w’, height ‘h’ and h*w is N, the number of pixels. In H each $\:{\text{X}}_{\text{k}}$ denotes one $\:{\text{k}}^{\text{t}\text{h}}$ band vector and mean of all image vectors can be computed as

1 $$\frac{1}{N}\sum\limits_{{i = 1}}^{N} {X_{i} }$$

where X_i_ is i^th^ band vector.

Consequently, the covariance matrix can be computed as

2 $$\:{\text{Covariance}} = \frac{1}{N}\sum\limits_{{i = 1}}^{{\text{N}}} {\left( {{\text{x}}_{{\text{i}}} - Mean} \right)\left( {{\text{x}}_{{\text{i}}} - Mean} \right)^{T} }$$

Correlation analysis is a statistically reliant practice and is used in Primary Partition as a part of band selection. This can be computed as

3 $$\:{\text{Correlation}}({\text{x}}_{{\text{i}}} ,{\text{x}}_{{\text{j}}} ) = \frac{{{\text{Covariance}}({\text{x}}_{{\text{i}}} ,{\text{x}}_{{\text{j}}} )}}{{\sqrt {{\text{Covariance}}({\text{x}}_{{\text{i}}} ,{\text{x}}_{{\text{i}}} ){\text{Covariance}}({\text{x}}_{{\text{j}}} ,{\text{x}}_{{\text{j}}} )} }}$$

Now, partition HSI as partition #1, partition #2… partition #i and each consists of $\:{l}_{1}$ ,$\:\:{l}_{2}$,……$\:\:{l}_{i}$ bands respectively such that

4 $$\sum\limits_{1}^{i} {l_{j} } = d$$

For each $\:{l}_{i}$ band group obtained from the Primary Partition based on correlation, secondary partition will be initiated. After coarser division, the neighborhood partition algorithm^23^ will be performed. Here $\:{m}_{1}$ ,$\:\:{m}_{2}$,……$\:\:{m}_{i}$ bands will be prioritized from each partition respectively.

After completion of the primary and secondary partition, we need to perform prioritization value to all the bands using wavelet entropy and then select the band with value in each partition to get the band subset. The wavelet oscillating function vanishes quickly and well in localization. The class of functions $\:\left\{{\text{f}}_{\text{a},\text{b}}\left(\text{x}\right)\right\}$ is generated by dilations and translations of the mother wavelet$\:{\upphi\:}\left(\text{x}\right)$. The wavelet function is defined as

5 $$\:{\text{f}}_{\text{a},\text{b}}\left(\text{x}\right)=\frac{1}{\sqrt{\text{a}}}\text{f}\left(\frac{\text{x}=\text{b}}{\text{a}}\right)$$

Here ${\text{a}},{\text{b}} \in {\text{R}}$called scale and shift coefficients. The continuous wavelet transforms of signal ${\text{S}}\left( {\text{x}} \right) \in {\text{L}}^{2} \left( {\text{R}} \right)$ (square-integrable real space). It can be represented as the correlation between $\:\text{S}\left(\text{x}\right)$ with the class of wavelet function. And it is the orthonormal basis for$\:\:{\text{L}}^{2}\left(\text{R}\right)$. Consequently, the energy concept comes into picture and can be defined as

6 $${\text{E}}_{{\text{j}}} = \sum\limits_{{\text{k}}} {\left| {{\text{C}}_{{\text{j}}} \left( {\text{k}} \right)} \right|^{2} }$$

Here each $\:{\text{C}}_{\text{j}}\left(\text{k}\right)$ denoted as wavelet coefficients of the correlated discrete wavelet transform. Further, the total energy is denoted as

7 $${\text{E}}_{{{\text{tot}}}} = \sum {{\text{E}}_{{\text{j}}} }$$

Then relative wavelet energy is (Here j represents resolution level)

8 $$\:{\text{P}}_{\text{j}}=\frac{{\text{E}}_{\text{j}}}{{\text{E}}_{\text{t}\text{o}\text{t}}}$$

Now wavelet entropy is defined as

9 $$\:{\text{S}}_{{{\text{wt}}}} = - \sum\limits_{{{\text{j}} < 0}} {{\text{P}}_{{\text{j}}} {\text{log}}[{\text{P}}_{{\text{j}}} ]}$$

Shannon entropy gives a measure of any distribution and a measure of degree of order/disorder is given by wavelet entropy. Also, it highlights the information of signal data on dynamical processes. Let be hyperspectral data after completion of the required band selection. Here prioritized bands are $\:{n}_{j}$ (j = 1, 2.,n) from each partition with

10 $$\sum\limits_{1}^{i} {n_{j} = p}$$

The total process is shown in Fig. 4.

### Bi-hemispherical reflectance recovery for spatial filtering

Generally, HSI data is peculiarized by its substrate surface properties. Bi-hemispherical reflectance is an intrinsic property of the surface that is based on the material properties of the earth, which is unfluctuating to imaging conditions and illumination. In the present work, Bi-hemispherical reflectance recovery is implemented as spatial filtering which is useful in the elicitation of semantic spatial information.

Bi-hemispherical reflectance recovery is applied on selected *p*-bands of $\:{{H}_{bs}\in\:\text{R}}^{\text{h}\times\:\text{w}\times\:\text{p}}$ HSI data. With the adjacent pixels $\:{x}_{m-1}\:and\:{x}_{m}$, the HSI transformed domain with isometric transformation to preserve the distance is shown in (11) and the first-order filter is given in (12).

Here, $\:{v}_{bs}\left[j\right]$ is the output corresponding to j^th^ input sequence ($\:{u}_{bs}\left[j\right]$) and feedback is $\:r\in\:\left[\text{0,1}\right]$. The transfer function is given in (13)

11 $$delta={\int\:}_{{x}_{m-1}}^{{x}_{m}}1+\frac{{s}_{p}}{{s}_{q}}*\left|{H}_{bs}^{{\prime\:}}\left(x\right)\right|\:dx$$

12 $$\:{v}_{bs}\left[j\right]=\left(1-b\right){u}_{bs}\left[j\right]+b{v}_{bs}\left[j-1\right]$$

Here, $\:{v}_{bs}\left[j\right]$ is the output corresponding to j^th^ input sequence ( $\:{u}_{bs}\left[j\right]$) and feedback is $\:r\in\:\left[{0,1}\right]$. The transfer function is given in (13)

13 $$\:{h}_{bs}\left[j\right]=(1-r){r}^{j}$$

The transfer function of $\:{h}_{bs}$ is given in (14). The neighbor pixel distance in transformed domain is delta.

14 $$\:{H}_{bss}\left[kk\right]=\left(1-{b}^{delta}\right){H}_{bs}\left[kk\right]+{a}^{delta}{H}_{bss}[kk-1]$$

Now, $\:{{H}_{bss}\in\:\text{R}}^{\text{h}\times\:\text{w}\times\:\text{p}}$ is the cube after spatial filtering.

### Classification using CNN

Prior to classification using CNN, we have implemented a non-linear version of PCA which involves a fuzzy variable in extracting the components. Then, CNN is used for carrying out the task of HSI classification. This consists of three major layers. The first layer is the convolution and process is given here under.

15 $$\:{\text{CONVO}}_{{{\text{i}},{\text{j}}}} = {\text{s}}\left( {\left( {F \otimes T} \right)_{{{\text{i}},{\text{j}}}} + b1} \right)$$

In this context, the symbol $\otimes$ represents the convolution operator, where* F* represents the filter, and $\:\left(\text{i},\text{j}\right)$ denotes the specific spatial location. T represents the input training vector derived from the tensor $\:{{H}_{bss}\in\:\text{R}}^{\text{h}\times\:\text{w}\times\:\text{p}}$ and $\:b1$ represents the bias term. The function σ (.) signifies the activation function. Specifically, in this study, ReLU is employed, as depicted below^16^.

16 $$\:{\upsigma\:}\left(x\right)=\text{m}\text{a}\text{x}(0,x)$$

The subsequent layer consists of pooling, which takes the feature maps obtained from the convolutional layer as its input. This model employs a maximum pooling layer to diminish the spatial dimensions of the feature maps, yielding$\:{\text{P}\text{O}\text{O}\text{L}}_{\text{i},\text{j}}$, as output. Following the alternating application of convolution and pooling layers, the feature maps transform into a flattened vector layout symbolized as FV. The third set comprises the Fully Connected Layers, tasked with extracting deep and abstract features.

17 $$\:\text{O}\text{V}=\:\sum\:{\upsigma\:}\left({\upomega\:}\text{*}\text{F}\text{V}+\text{b}2\right)$$

In the present context, σ (.) represents the activation function. The soft-max function is employed at the final layer, while the remaining layers utilize ReLU as an activation function.$\:\:b2$ is bias, OV denotes the output vector and $\:{\upomega\:}$ weight.

### Datasets

**Fig. 5:**
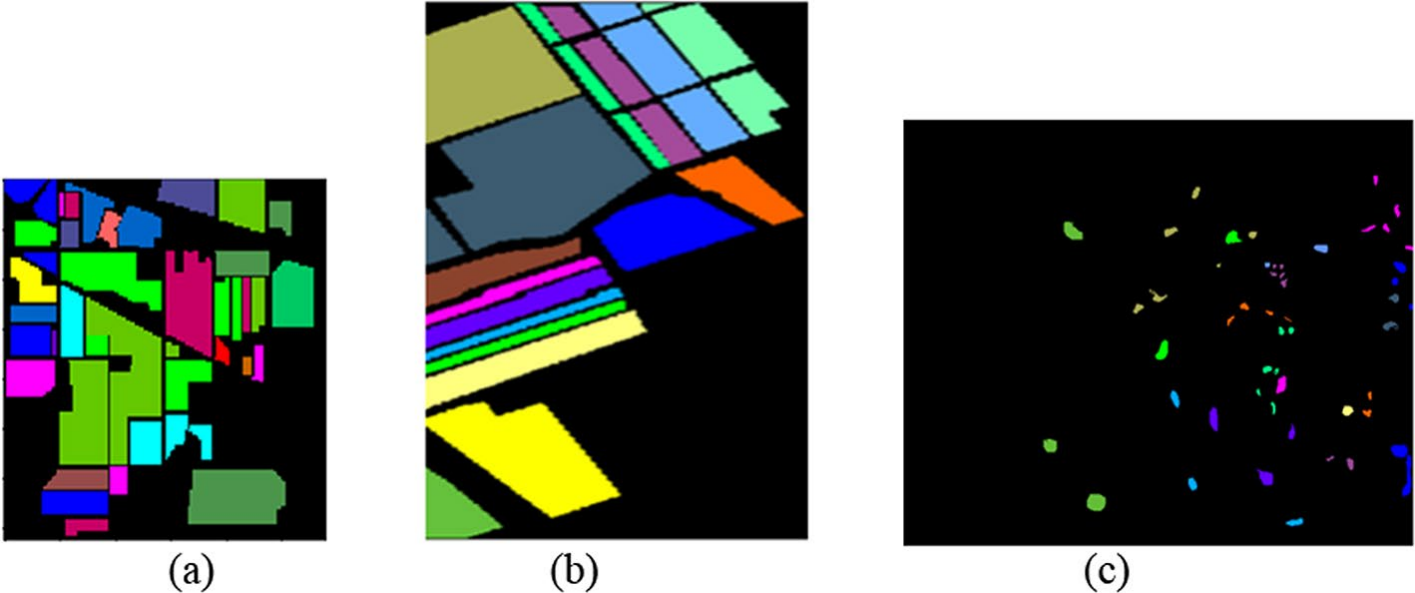
True image (**a**) Indian_Pines (**b**) Salinas (**c**) KSC.

The datasets along with the ground truth used are given in Table 1; Fig. 5 respectively.

**Table 1:** Description of three Hyperspectral datasets.

Datasets	Indian Pines	Salinas	KSC
Number of Pixels	145*145	512*217	614*512
Number of Bands (Post_Denoising bands)	224 (200)	224 (204)	224 (176)
Number of classes	16	16	13
Number of Labeled pixels	10,249	54,129	5,211
Wavelength	400–2500 nm	400–2500 nm	400–2500 nm
Sensor (Acquisition Year)	AVIRIS (12-June-1992)	AVIRIS (09-October-1998)	AVIRIS (23-March-1996)
Spatial resolution	20 m	3.7 m	18 m
Area	Indiana, USA	California, USA	Kennedy_Space_Center, USA

## Results and analysis

This section analyzes the efficiency of the selected bands and the proposed approach to the datasets.

### Classification accuracies and class-wise accuracies

The performance of the approach is tested with the following methods: firstly, UBS An unsupervised band selection method leveraging a multimodal evolutionary algorithm and subspace decomposition is the first comparative method^49^. This approach initially utilizes the multimodal evolutionary algorithm within spectral subspace decomposition to uncover multiple global or local solutions, thus boosting the diversity of band subsets. It also emphasizes the ordered property by increasing the distinction between neighboring band subspaces to minimize redundancy. Experimental results on three widely used hyperspectral remote sensing datasets and a collected composition prediction dataset showcase the method’s effectiveness. The second method is FDCNN^73^ is used to compare the results. Here, the method has achieved overall accuracies of 96.8% and 98.9% on Indian pines and Salinas. Thirdly, a method with WEDCT-MI^35^ is used for comparison. Here, weighted entropy is the measure. The method results in an overall accuracy of 84.6 and 99.1% on the Indian pines and Salinas dataset. Fourthly, Hybrid-CR clustering-based method is used to test the results^51^. This method results in the overall accuracy of 93.8% and 92.8% on the Indian pines and Salinas datasets respectively.

The last comparative method used is BS-MO^11^ in which a multi-objective approach is used for band selection.

**Table 2:** Comparison of classification accuracies (%).

Class name	UBS	FDCNN	WEDCT-MI	Hybrid-CR	BS-MO	Proposed Method
Alfalfa	98.5	100	69.44	98	91.67	100
Corn-notill	99	93.03	78.98	89	99.16	99.4
Corn-mintill	98.3	99.58	75.60	92	98.08	100
Corn	99	71.33	66.67	98.5	96.67	100
Grass-pasture	99	98.69	89.64	97.6	98.35	99.7
Grass-trees	98	99.9	97.09	100	99.45	100
Grasspasturemowed	98.6	77.79	81.88	98.12	98	100
Soybean-notill	96	93.71	81.60	93.5	97.12	99.55
Oats	98.5	99.99	56.13	96.6	98.44	100
Soybean-mintill	98.6	99.11	82.29	88.56	98.05	99.7
Haywindrowed	99	99.99	98.79	99	99	100
Soybean-clean	99.6	96.55	74.47	95	97.32	99.03
Wheat	98.4	100	92.07	99	99	99.3
BuildingsGrass-TreesDrives	93	97.76	65.56	98.46	96.90	99.99
Woods	100	100	96.44	100	99.68	100
Stone-Steel-Towers	98	98.48	94.61	99.99	97.99	100
Overall Accuracy (OA)	98	96.87	84.61	93.85	96.9	**99.72**
Average Accuracy (AA)	98.21	95.37	81.33	96.46	97.81	**99.79**
κ	97.99	96.43	82.41	93	97.03	**99.68**

**Fig. 6:**
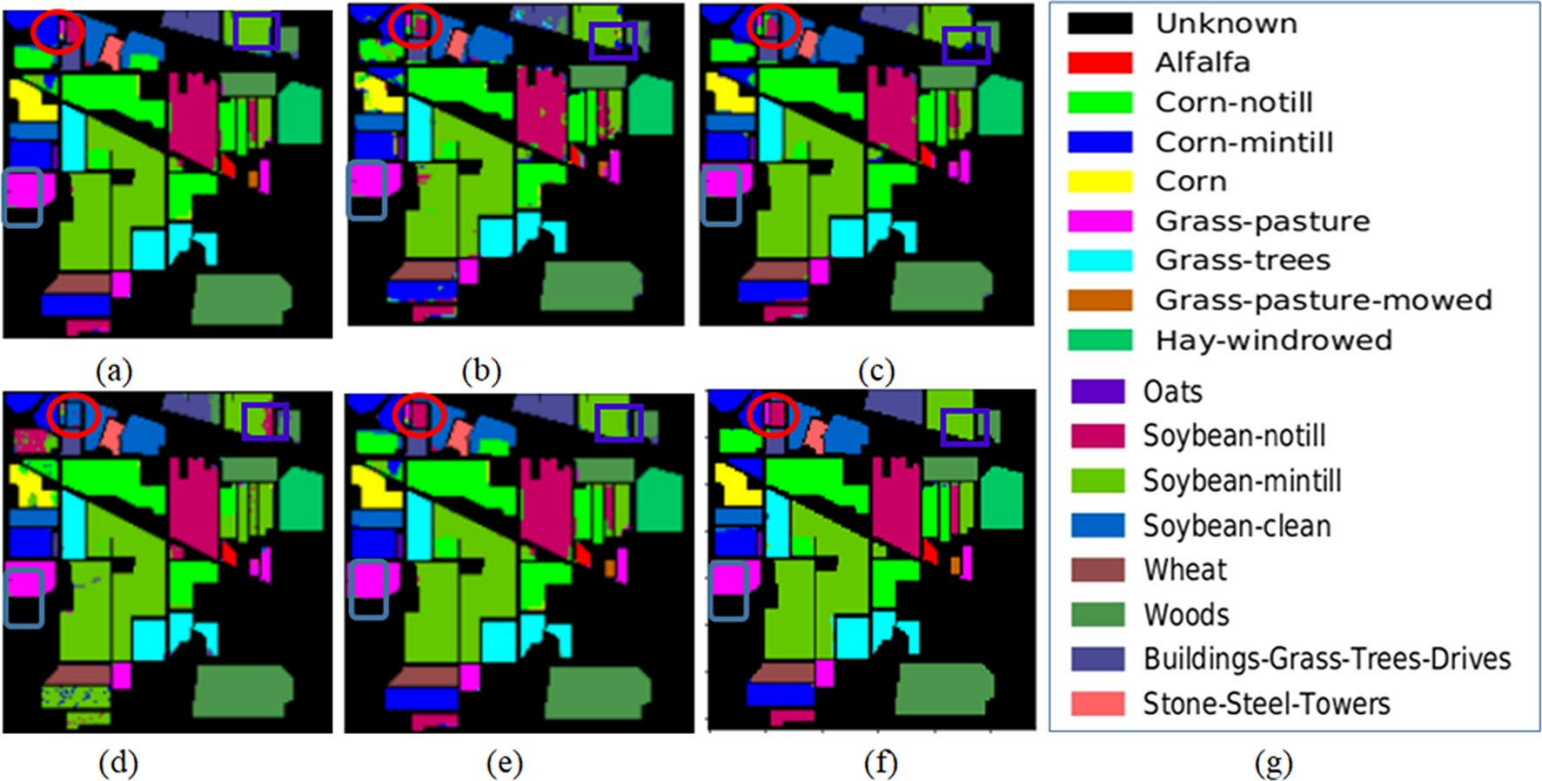
Maps on Indian Pines (**a**) UBS (**b**) FDCNN (**c**) WEDCT-MI (**d**) Hybrid-CR (**e**) BS-MO (**f**) Proposed method (**g**) Category label.

**Fig. 7:**
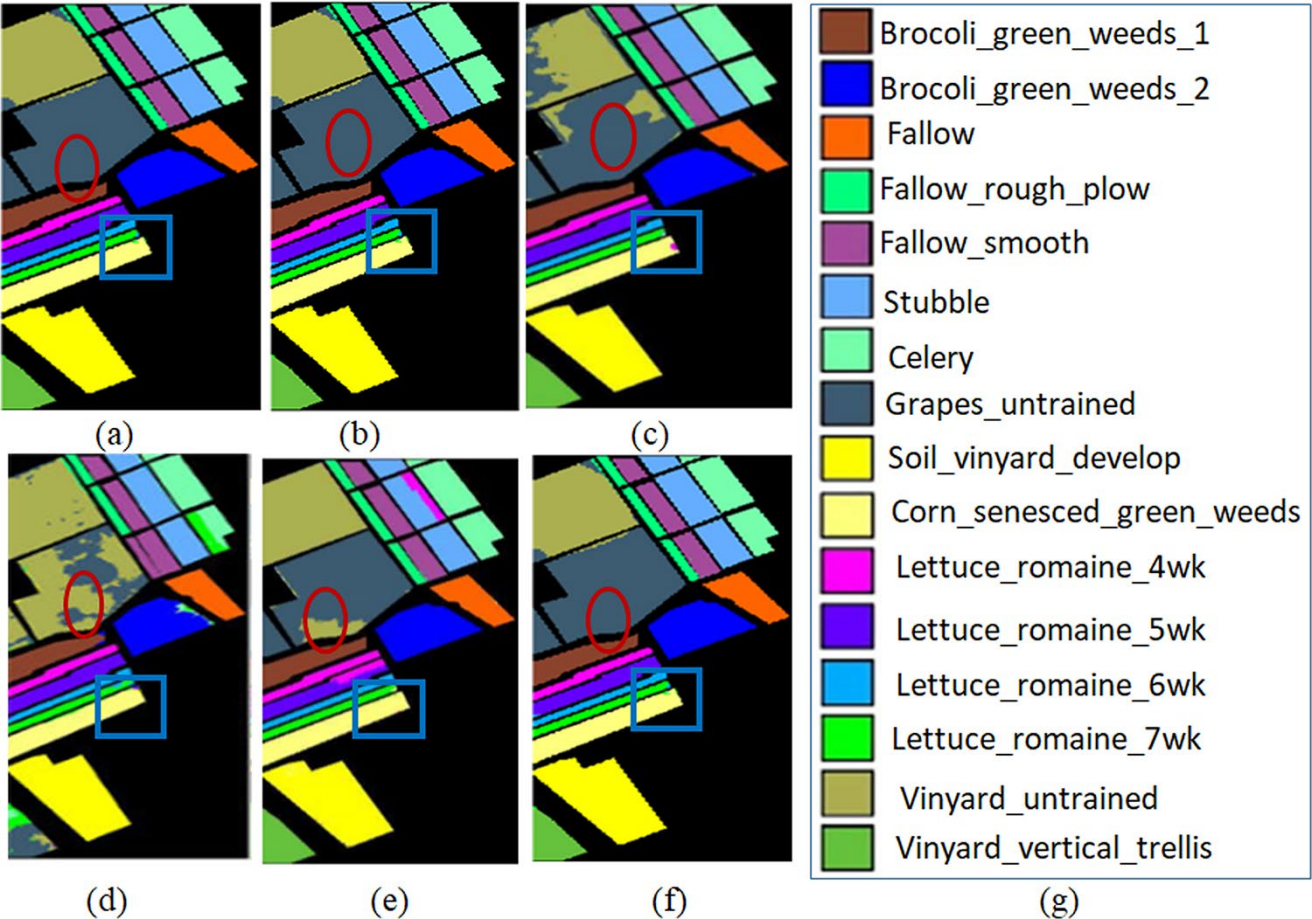
Maps on Salinas (**a**) UBS (**b**) FDCNN (**c**) WEDCT-MI (**d**) Hybrid-CR (**e**) BS-MO (**f**) Proposed method (**g**) Category label.

**Fig. 8:**
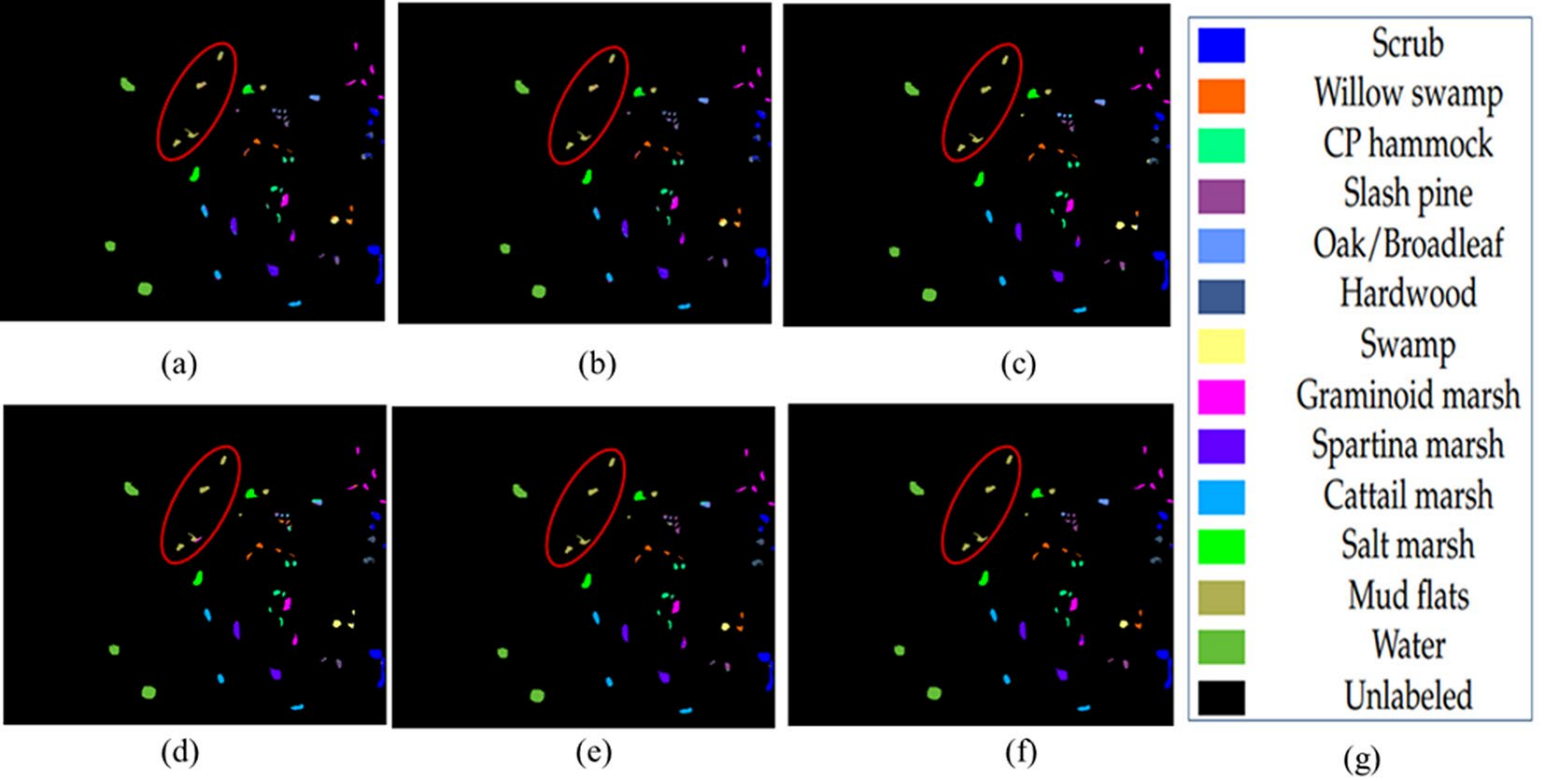
Maps on KSC (**a**) UBS (**b**) FDCNN (**c**) WEDCT-MI (**d**) Hybrid-CR (**e**) BS-MO (**f**) Proposed method (**g**) Category label.

The qualitative maps on Indian Pines, Salinas and KSC datasets are shown in Figs. 6, 7 and 8 respectively. The classes with en-circled and en-rectangled show the superiority of the proposed method on datasets.

Tables 2 and 3 provide the accuracy measures of proposed method with other competitive methods on Indian Pines and Salinas respectively.

**Table 3:** Comparison of classification accuracies (%) obtained by the proposed framework with State-of-the-art methods: Salinas dataset.

Class name	UBS	FDCNN	WEDCT-MI	Hybrid-CR	BS-MO	Proposed Method
Brocoligreenweeds_1	98	99.99	99.61	98.89	98.61	99.7
Brocoligreenweeds_2	99	99.99	99.97	99.01	99.07	99.01
Fallow	97.34	99.90	99.64	99.06	95.79	98.4
Fallow_roughplow	98.67	95.96	99.58	100	99.2	99
Fallow_smooth	98.54	99.78	99.03	98	96.26	99
Stubble	100	100	99.88	98.5	99.53	99.6
Celery	98.79	99.94	99.71	100	99.47	99.7
Grapes_untrained	99	97.33	89.38	94.3	86.71	98.8
Soilvinyarddevelop	89.66	99.86	99.88	97.9	98.98	98
Cornsenesced_greenweeds	96.99	99.99	95.96	97	93.08	99
Lettuce_romaine_4wk	100	99.82	99.18	98.98	95.21	99.1
Lettuce_romaine_5wk	99	99.89	99.82	99.01	99.63	99.8
Lettuce_romaine_6wk	98	97.10	99.43	99.81	97.36	99.7
Lettuce_romaine_7wk	99	95.98	98.52	99.8	93.99	98.9
Vinyard_untrained	99	98.19	76.49	93.86	68.45	100
Vinyard_vertical_trellis	97.98	99.99	99.07	98.9	97.43	100
Overall Accuracy (OA)	98.99	98.92	99.14	97.13	91.56	**99.94**
Average Accuracy (AA)	98.06	98.98	97.20	98.31	94.92	**99.23**
κ	98.1	98.81	93.43	96.82	90.57	**98.03**

**Table 4:** Comparison of classification accuracy (%) obtained by the proposed framework with State-of-the-art methods: KSC dataset.

Class name	UBS	FDCNN	WEDCT-MI	Hybrid-CR	BS-MO	Proposed Method
Scrub	89.89	88.67	98.4	96.86	99.89	99.89
Willow	70.2	74.02	89.1	59.02	87.15	89.15
CP hammock	65.8	66	86.88	76.56	89.33	89
CP/Oak	54.52	50.45	91.89	38.1	93.67	92.99
Slash pine	64.23	65	95.97	80.49	92.12	97.12
Oak/Broadleaf	67.34	69.49	95.99	79.31	96.45	97.89
Hardwood swamp	76.45	77.34	98.77	84.62	100	100
Graminoid marsh	86.44	83.88	93	83.33	89.66	94.98
Spartina marsh	78.45	77.67	99.02	95.38	98.44	99.48
Catiail marsh	95.88	97.3	87.1	49.5	87.34	89.78
Salt marsh	97.2	98	98.6	99.05	100	100
Mud flats	92.74	94.39	96.33	77.78	97.25	99.25
Water	97	98.66	98.55	96.55	100	100
**Overall Accuracy (OA)**	85.38	85.65	95.67	83.07	96.05	**97.23**
**Average Accuracy (AA)**	79.9	80.06	94.58	78.19	94.71	**96.11**
**κ**	84.67	84.96	95.44	82.42	95.85	**97.08**

The proposed method can effectively identify the different crop classes on both the datasets Indian Pines, Salinas, and KSC datasets. There exist two types of crops (corn and soy) from the Indian Pines dataset. The accuracy values obtained for the crop classes are compared across the state-of-the-art methods as shown in Table 5. Corn crop classes like Corn-notill, Corn-mintill, and Corn with class numbers 2, 3, and 4 have achieved AA in the range of 73.75-99.8%. Soy crop classes like Soybean-no-till, Soybean-min-till, and Soybean-clean with class numbers 10, 11, and 12 have reported 79.45-99.42% as the accuracy range. There are two types of crops (Fallow and Lettuce) from the Salinas dataset. The accuracy obtained for the crop classes is compared across state-of-the-art methods. Fallow crop classes like Fallow, Fallow_rough_plow and Fallow_smooth with class numbers 3, 4, and 5 have achieved AA in the range of 98.55-99.42%. Lettuce crop classes like Lettuce_romaine_4wk, Lettuce_romaine_5wk, Lettuce_romaine_6wk, and Lettuce_romaine_7wk with class numbers 11, 12, 13, and 14 have reported 98.19-99.37% as the accuracy range. These outperforming values support the fact of applying models for crop classification applications.

The classification accuracies on the KSC dataset with the state-of-the-art methods are shown in Table 4. It is shown that the classes Willow, Slash pine, Oak, Graminiod marsh, Spartina Marsh, Catiail_march, Mud flats, and Water have shown good accuracy when compared with other class accuracies. Also, the reported OA is 97.23% whereas the methods show between 83% and 96.05%. This indicates that the Wavelet-based approach with spatial feature extraction efficiently extracts the features relevant to the classes. Also, the reported AA is 96.11% with the proposed approach and it ranges from 79.9 to 94.7% with the other methods. Qualitatively, the classification maps of the KSC dataset are given in Fig. 8. As an example, the class Mudflats has shown good classification when compared to other methods.

### Selected bands from the competing methods

Table 5 shows the band subsets selected by the six BS methods from the literature along with proposed method for the Indian Pines, Salinas HSI and KSC datasets. Though the number of bands are less when compared with the other methods, the proposed methods due to their dual partitioning results in high classification accuracy and the mean spectral divergence is also having higher value.

**Table 5:** Bands selected from competing methods.

Method	Indian Pines	Salinas	KSC
UBS	19,28,44,118,134,160,56,87,161,162,163	16, 32, 46, 94, 122, 164, 165,166,180	2,10,13,22,29,33,36,40, 47,50,55,69,77,90,98
FDCNN	2,17,19,36,46,47,58,66,72,74,75,76,80,101,198	1,11,18,21,24,30,35,38,39,40,41,43,44,45,47,52,53,180	6, 9, 15, 18, 28, 32, 36, 43,52, 63,69, 70,71,74, 77,106,122,149,165,171
WEDCT-MI	9,13,14,23,31,32,38,44,51,54,60,75,79,94,95,96,97,110,127,141,150,155,156,166,167,180,181,188,191,196	8,9,10,12,14,15,21,25,27,31,33,34,36,40,43,47,55,58,60,64,68,75,76,83,85,87,93,97,135,186	1,6, 15, 26, 39,43, 63, 77, 115, 138, 149, 171
Hybrid CR	3, 10, 16, 44, 67, 72, 77, 99, 114, 131, 149, 162, 172, 178	6, 14, 19, 30, 44, 56, 70, 103, 113, 118, 133, 159, 167, 176, 185, 194	1, 13, 20, 25, 32, 36, 40, 46, 51, 56, 59, 79,91,100,133
BS MO	2, 5,10, 16,24, 26, 27, 29, 36, 41, 53, 60, 67,72, 74,77, 99,108,113,131,149,162, 172,178,185	6,14,19,27,34,44,56,62,66,74,81,92,104, 113,117, 126,132,137,157,165,176,182,190	1, 28, 45, 51, 71, 75, 100, 107, 133, 134, 160, 174
Proposed	1,35,59,63,67,68,69,70,72,94,95,200	1,84,106,127,128,40,146,147,148,149,150,151,152,157,204	5, 16, 24, 45, 49, 69, 78, 97, 117, 128, 152, 155

### Spectral reflectance curves of the selected bands

**Fig. 9:**
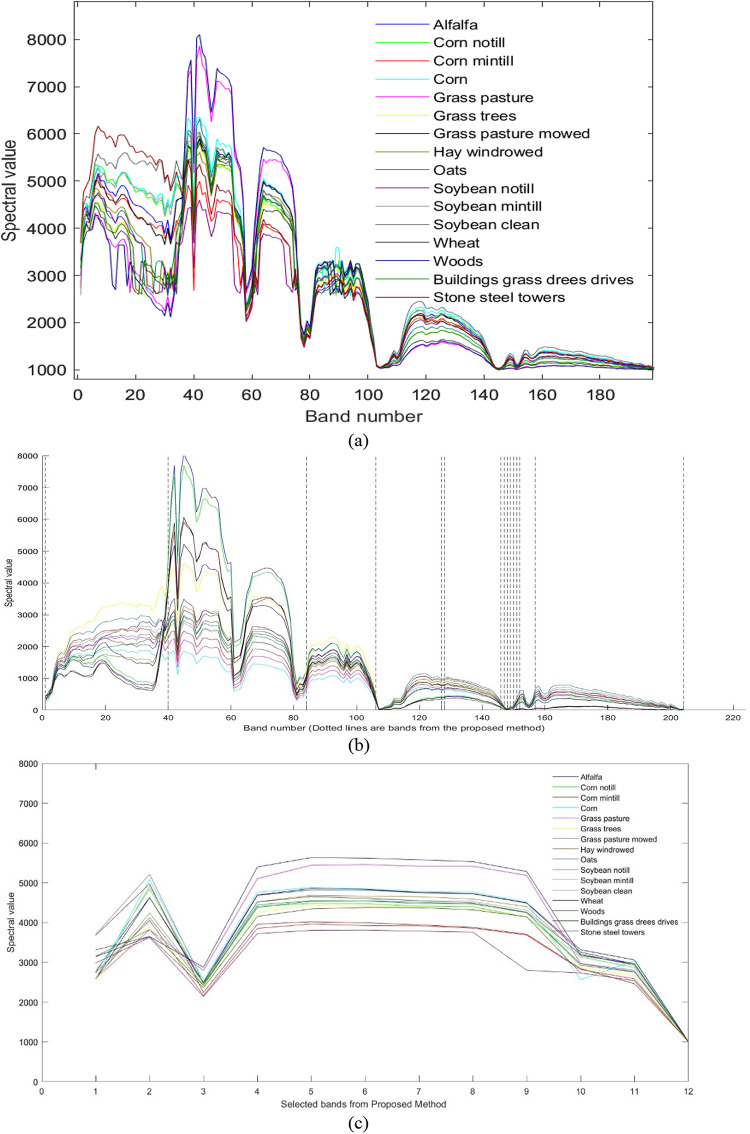
Spectral Responses on Indian Pines (**a**) Original data set (**b**) Selected bands (**c**) Responses of the selected bands.

**Fig. 10:**
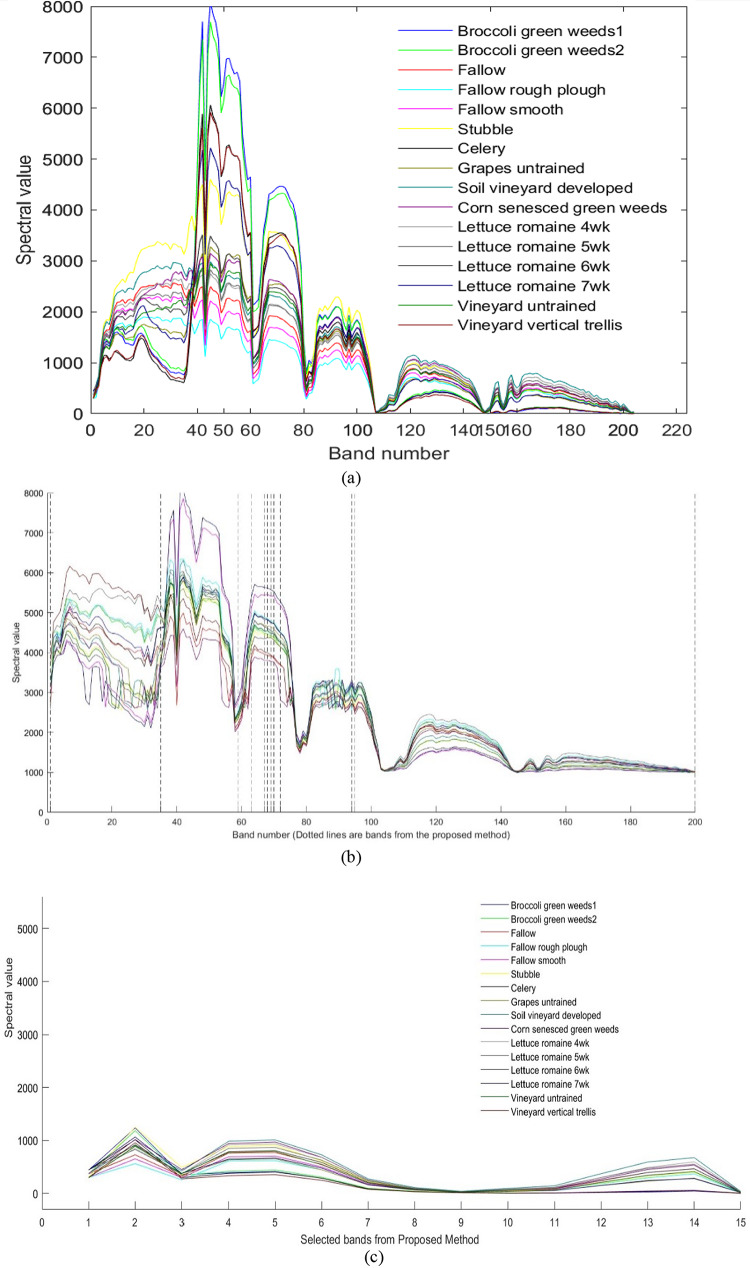
Spectral Responses on Salinas (**a**) Original data set (**b**) Selected bands (**c**) Responses of the selected bands.

**Fig. 11:**
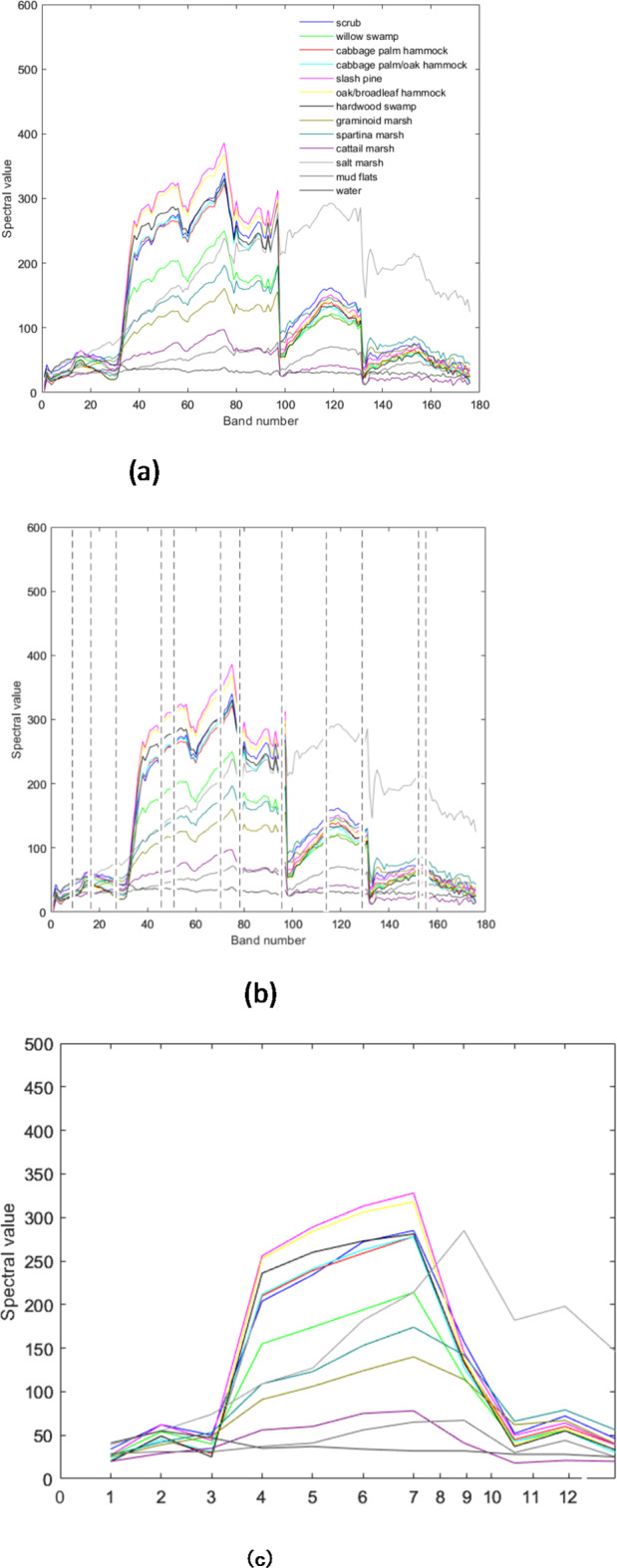
Spectral Responses on KSC (**a**) Original data set (**b**) Selected bands (**c**) Responses of the selected bands.

The spectral reflectance curves of the selected bands on Indian Pines, Salinas, and KSC datasets are shown in Figs. 9, 10 and 11 respectively. It can be observed from Fig. 9(a) and (c) that the spectral response is similar to the original dataset. It indicates that the selected Indian Pines dataset has the same spectral response as the original bands. Figure 9(b) highlights the band selected with the proposed method. Similarly, Fig. 10(a) and (c) indicate that the spectral responses of the selected band set are the same as the original band’s response. Also, Fig. 10(b) contains the bands selected from the proposed method. From the figures, it can be understood that the bands selected have the same spectral response as the original one. The spectral responses of the KSC dataset with the selected bands, original responses, and the responses embedded in the dataset are shown in Fig. 11.

It can be observed that the spectral responses of the selected bands are like the spectral responses of the original ones, which indicates that the selected bands have the same amount of information as in the original ones.

### Comparative analysis of the selected bands with the state-of-the-art methods

#### Analysis of selected bands via mean spectral divergence

**Fig. 12:**
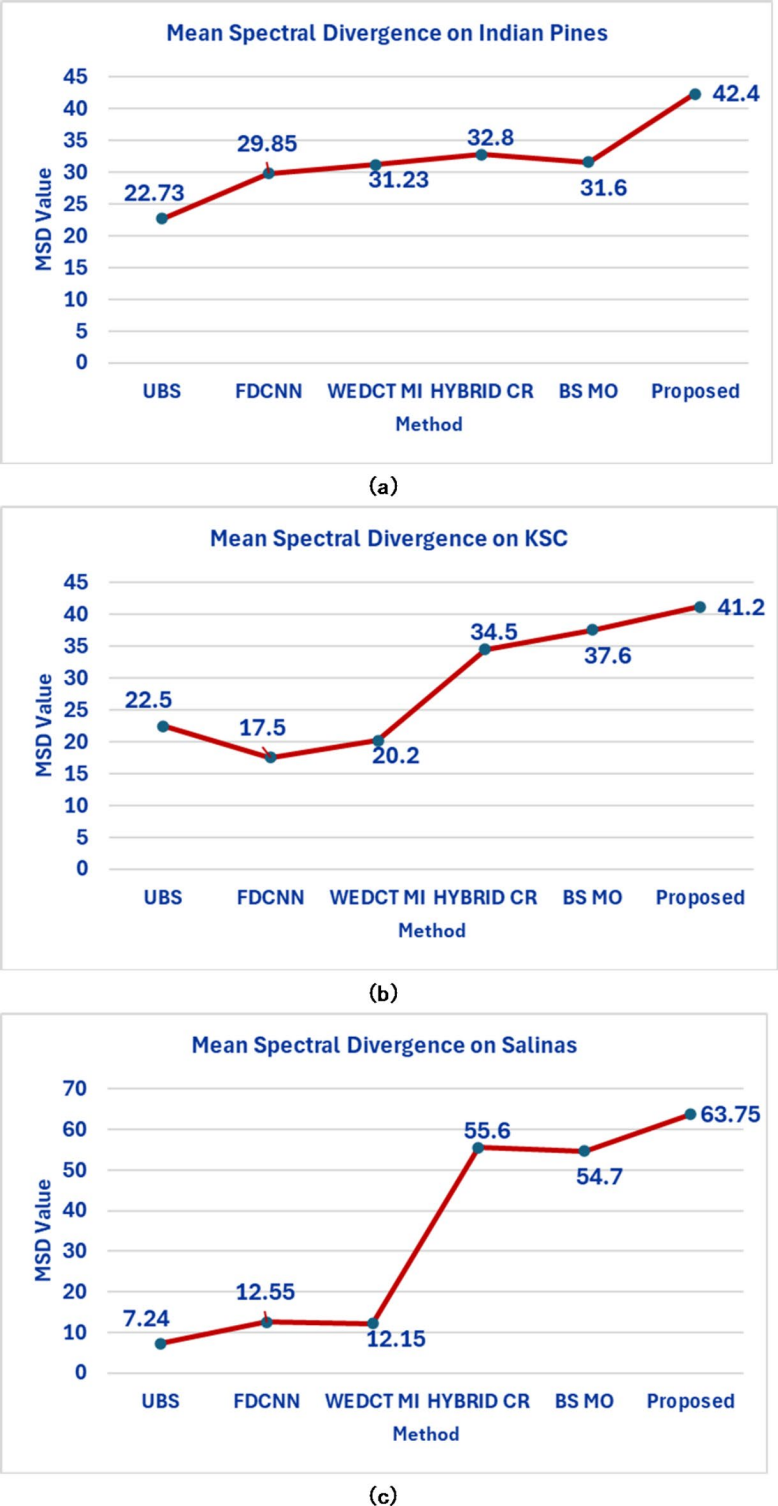
Mean Spectral Divergence of selected bands (**a**) Indian Pines (**b**) Salinas (**c**) KSC.

The Mean Spectral Divergence (MSD), an assessment statistic, is also used in the present work to analyze the effectiveness of the chosen bands. This measure is used to evaluate the spectral bands’ redundancy. Based on the grey histogram between the bands, MSD is calculated. The mean spectral divergence (MSD) is calculated via Eq. (18).

18 $${\text{MSD}}\left( S \right) = \frac{1}{{n(n - 1)}}\sum\limits_{{i = 1}}^{n} {\sum\limits_{{j = 1}}^{n} {D_{{SKL}} } } \left( {B_{i} ||B_{j} } \right)$$

where $\:{D}_{SKL}$ is the symmetrical Kullback–Leibler divergence which measures the dissimilarity between bands $\:{B}_{i}$ and $\:{B}_{j}$. Lower redundancy in the chosen bands is indicated by higher MSD values, and vice versa. From Fig. 12(a), (b), and (c) for Indian Pines, Salinas, and KSC datasets. The proposed method has an advantage when using multiple partitions with the number of bands ranging from 8 to 20. In particular, the proposed algorithm performs better than others by selecting 12 bands from Indian Pines, KSC data, and 15 bands from Salinas data respectively with higher MSD values. The MSD values are 42.4, 63.75, and 41.2 on Indian Pines, Salinas, and KSC datasets respectively which is higher in comparison with other methods. This indicates that the proposed method selected the bands, which are divergent and helped to find the classes effectively during the classification.

#### Analysis of selected bands on the state of art methods via entropy

**Fig. 13:**
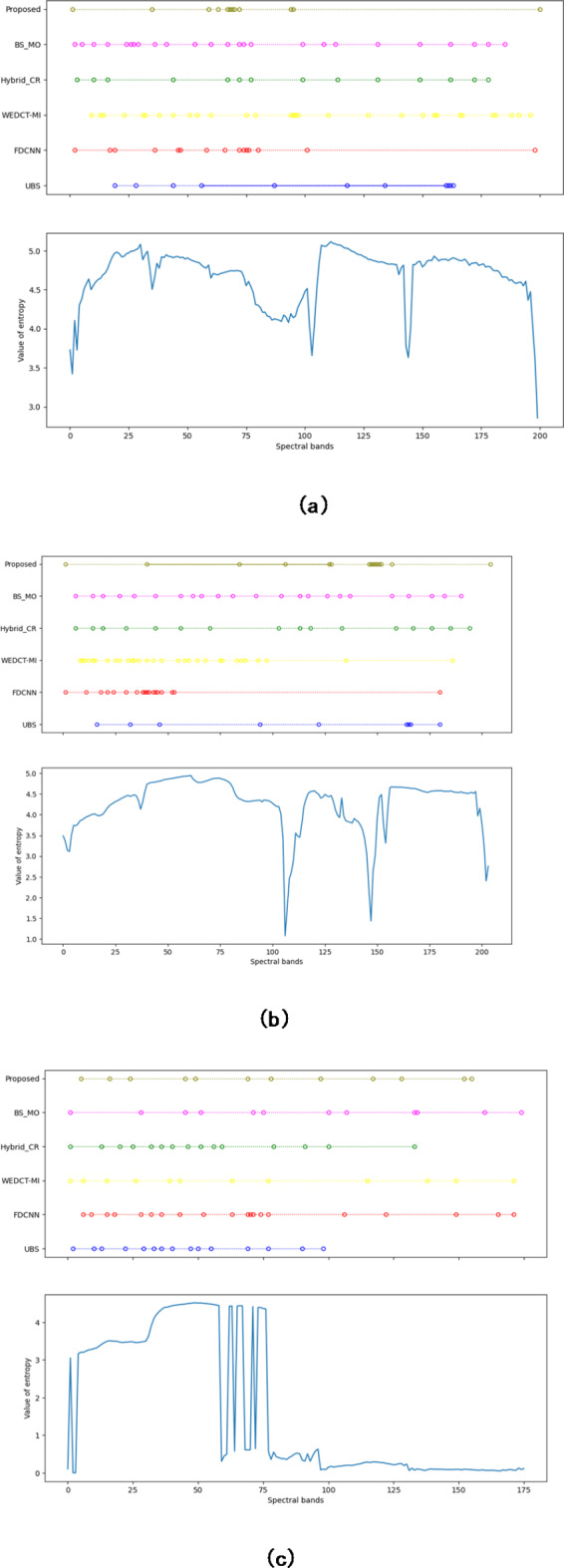
Entorpy of the selected bands on the datasets (**a**) Indian Pines (**b**) Salinas (**c**) KSC.

To know the effectiveness of the proposed methods on the selected bands, entropy measures are computed. The entropy will specify how much information a particular band holds. The entropy of the selected bands from each of the methods and the entropy of all the bands of the datasets (Indian Pines, Salinas, and KSC) are shown in Fig. 13. On the Indian Pines dataset, the entropy of the selected bands from all the methods is shown in Fig. 13(a). Figure 13(a) comprises of top and bottom figures. The top figure indicates the selected bands from all the methods, bottom figure indicates the entropy of each of the bands from the dataset. Here, the band selected is represented by a circle. The respective entropy can be seen at the bottom figure. It is observed that on the Indian Pines, the selected bands have the highest entropy compared with all other methods. As an example, the 28th band of the Indian Pines data set has higher entropy and is selected by the proposed method. Similarly, the 163rd band also has higher entropy. This indicates the proposed method selects the band which has the highest entropy. Similarly, on the Salinas dataset, the entropies of the selected band set from all the methods and each of the original datasets is presented in Fig. 13(b). It can be observed that the proposed method selects the bands which have higher entropy. As an example, band 146 of Salinas’s higher entropy was selected by the proposed method. Similarly, Fig. 13(c) depicts the entropies of selected bands and the original KSC data set. As an example, the bands 49 and 69 have higher entropy and are selected by the proposed method. This showcases the efficiency of the proposed method.

### Sensitivity analysis

#### Sensitivity concerning different bands

**Fig. 14:**
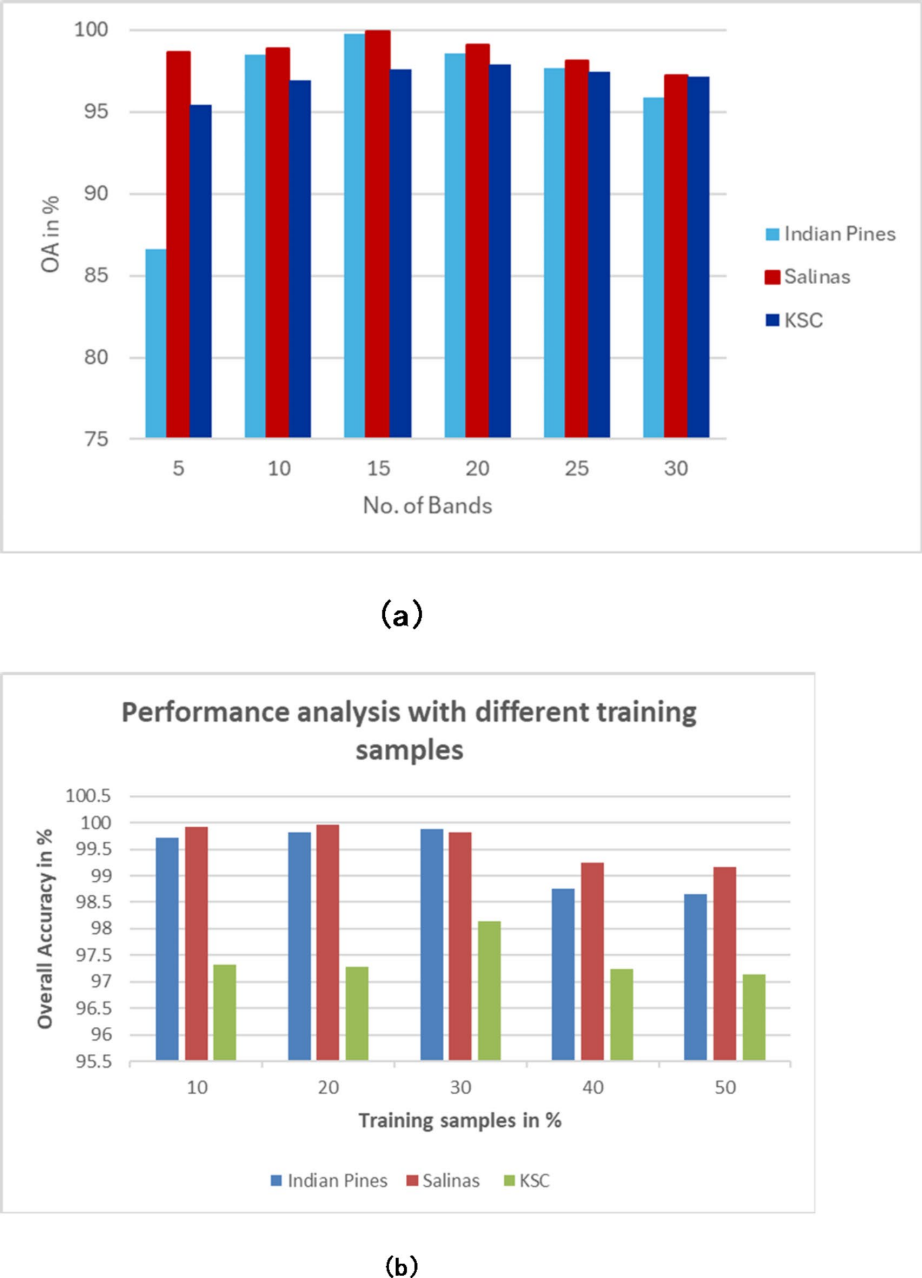
(**a**) Overall accuracy curves of Indian Pines, Salinas, and KSC datasets. (**b**) Performance analysis with different set of training samples.

To test the efficacy of the methods, experiments are conducted by varying the number of dimensionality bands on three datasets. From each dataset, selected 5,10, 15, 20, 25, and 30 bands and then performed the classification and reported the accuracy. Figure 14(a) depicts Overall Accuracy values on three datasets. As it is observed that the accuracy is reduced as the number of bands increases. The accuracy ranges between 86.65% and 99.76% on Indian Pines, 98.7% and 99.90% on Salinas, and 95.43% and 97.9% on KSC datasets for the respective band set which ranges from 5 to 30 bands.

#### Sensitivity to different training samples

Experiments are also conducted to test sensitivity with different training samples. We have selected 10%, 20%, 30%, 40% and 50% of training samples and conducted the classification. The results are depicted in 14(b). The classification accuracy is decreased as and when the number of training samples increases (Hughes phenomenon). On Indian Pines data, the accuracy ranges between 98.65 and 99.89%, on Salinas, OA ranges between 99.17 and 99.96% and on KSC data, OA ranges between 97.14 and 98.14%.

#### Analysis of the crop classes over the datasets

To test the performance of the proposed state-of-the-art methods, the efficacy concerning crop classes on the three datasets also experiments and the results are provided in Table 6. The proposed model is evaluated for different crops and vegetation types from Indian Pines such as Corn and Soy classes, the AA values are reported as 99.8% and 99.42% respectively. On the Salinas dataset, the classes Fallow and Lettuce category reported an AA of 98.8% and 99.37% respectively. Similarly, on the KSC dataset, three subcategories Forests and Woodlands, Wetlands and Marshes, and Scrub are identified and reported with an AA of 95.4%, 94.68%, and 89.89% respectively.

**Table 6:** Comparison of the various crop class results across the models.

Dataset	Crop Type	Class name (number)	UBS	FDCNN	WEDCT-MI	Hybrid-CR	BS-MO	Proposed Method
**Indian Pines**	**Corn**	Corn-notill(2)	99	93.03	78.98	89	99.16	99.4
		Corn-mintill(3)	98.3	99.58	75.60	92	98.08	100
		Corn(4)	99	71.33	66.67	98.5	96.67	100
		AA	98.76	87.98	73.75	93.16	97.97	99.8
	**Soy**	Soybean-notill(10)	98.6	93.71	81.60	93.5	97.12	99.55
		Soybean-mintill(11)	99	99.11	82.29	88.56	98.05	99.7
		Soybean-clean(12)	99.6	96.55	74.47	95	97.32	99.03
		**AA**	**99.06**	**96.45**	**79.45**	**92.35**	**97.49**	**99.42**
**Salinas**	**Fallow**	Fallow(3)	97.34	99.90	99.64	99.06	95.79	98.4
		Fallow_rough_plow(4)	98.67	95.97	99.59	100	99.2	99
		Fallow_smooth(5)	98.54	99.78	99.03	98	96.26	99
		**AA**	**98.18**	**98.55**	**99.42**	**99.02**	**97.08**	**98.8**
	**Lettuce**	Lettuce_romaine_4wk (11)	100	99.81	99.19	98.97	95.21	99.1
		Lettuce_romaine_5wk (12)	99	99.90	99.80	99	99.63	99.8
		Lettuce_romaine_6wk (13)	98	97.08	99.44	99.8	97.36	99.7
		Lettuce_romaine_7wk (14)	99	95.99	98.51	99.8	93.99	98.9
		**AA**	**99**	**98.19**	**99.2**	**99.3**	**96.54**	**99.37**
**KSC**	**Forests and Woodlands**	CP hammock	65.8	66	86.88	76.56	89.33	89
		CP/Oak	54.52	50.45	91.89	38.1	93.67	92.99
		Slash pine	64.23	65	95.97	80.49	92.12	97.12
		Oak/Broadleaf	67.34	69.49	95.99	79.31	96.45	97.89
		Hardwood swamp	76.45	77.34	98.77	84.62	100	100
		**AA**	**65.67**	**65.66**	**93.90**	**71.82**	**94.31**	**95.40**
	**Wetlands and Marshes**	Willow	70.2	74.02	89.1	59.02	87.15	89.15
		Graminoid marsh	86.44	83.88	93	83.33	89.66	94.98
		Spartina marsh	78.45	77.67	99.02	95.38	98.44	99.48
		Catiail marsh	95.88	97.3	87.1	49.5	87.34	89.78
		Salt marsh	97.2	98	98.6	99.05	100	100
		**AA**	**85.63**	**86.17**	**93.36**	**77.26**	**92.52**	**94.68**
	**Scrub**	**AA**	**89.89**	**88.67**	**98.4**	**96.86**	**99.89**	**99.89**

### Strengths and weaknesses of proposed methodology

The method effectively reduced the redundancy and prioritized the bands via correlation and wavelet approaches to retain essential spectral data which helps to improve the interpretability of data. Also, focused on neighborhood partition and albedo recovery to make use of spectral-spatial consistency. The effectiveness of the proposed method is assessed with different strategies as follows:

- The quantitative analysis via individual class-wise accuracies and the performance measures such as Overall Accuracy, Average Accuracy, and Kappa Coefficient. These accuracies are shown in Table 2 for Indian Pines, Table 3 for Salinas, and Table 4 for the KSC dataset.
- The quality of the proposed approach is tested via classification maps. The classification maps of all the methods are given in Fig. 6 for Indian Pines data, Fig. 7 for Salinas, and Fig. 8 for KSC datasets.
- The performance of the proposed band selection is also tested with Mean Spectral Divergence(MSD) and entropy. The MSD specifies how the selected bands are divergent from each other so that redundancy will be reduced. The entropy will provide information about how much information exists in the selected bands on each of the selected bands. The MSD computational values and Entropies of the state-of-the-art methods are shown in Figs. 12 and 13 respectively.
- Also, to implement the proposed approach for crop classification problems, Table 6 presents how the algorithm is effective in classifying various crop classes and their accuracies. The crop classes’ performance is compared with state-of-the-art methods.
- To test the efficacy of the selected bands, sensitivity analysis is also performed by varying different numbers of bands over the three datasets, and the results are reported. Also, by changing the different number of training samples, the performance of the proposed approach is tested. Figure 14(a) shows the sensitivity concerning the different number of bands selected from the proposed band selection. Figure 14(b) shows the sensitivity with respective to different number of training samples.

With all these, it is observed that the proposed band selection approach followed by CNN-based classification helps to improve the classification accuracy over the competitive approaches.

One of the limitations of the proposed approach is the manual selection of the parameters. The proposed method contains tuning parameters in different stages like Dual Partitioning, Band Prioritization, and spatial filtering. The key parameters in the dual partitioning stage include the number of partitions, correlation threshold, and neighborhood size. The number of partitions was set based on an initial analysis of the dataset, allowing for stability between sufficient coarseness and computational efficacy. The correlation threshold used in the correlation analysis was pragmatically determined by experimenting with values ranging from 0.4 to 0.9, with attention to maximizing the engaged informative bands and minimizing redundancy. According to Eq. 3, the threshold value is set as 0.6. Finally, the neighborhood size parameter, which commands the extent of local context carefully in secondary partitioning, was fixed through a grid search, leading to a size of five pixels created based on its influence on band prioritization. For the Band Prioritization stage, the key parameters involved according to Eq. 5 are scale and shift coefficients and levels of resolution in wavelet decomposition. The scale and shift coefficients were analytically changed and set to 2 and 1, respectively. These were found to maintain a good balance in computational intricacy. The resolution levels were set to 3, which allowed for a complete analysis of the wavelet energy according to Eq. 8. To enumerate the implication of each band, wavelet entropy was calculated according to Eq. 9, with thresholds set according to sensitivity analysis, ensuring that bands paying high entropy values were prioritized successfully. In our feature work, we will adaptively tune the parameters in an unsupervised manner without compromising the classification accuracy.

### Theoretic and running time complexity

This section describes the time complexity analysis (both theoretical and running). The grouping of bands with M bands and entropy computational costs are O(2M^2^) and O(M). The cost of selection of m bands is O(m). Hence, the band selection time complexity is X = O(2M^2^ ) + O(M) + O(m). Bi-hemispherical reflectance recovery for spatial filtering with pixels is $\:\varvec{Y}=\varvec{O}\left(\varvec{p}\varvec{*}\varvec{q}\right)$. The CNN complexity is ${\rm Z}=\rm O\sum\limits_{l=1}^{\rm nc}(n_{l-1}{s_{l}^2}n_{l}{m_{l}^2})$ here, l convolution layer index, total convolution layers, nc, total filters are $\:{\mathbf{n}}_{\mathbf{l}}$, $\:{\mathbf{s}}_{\mathbf{l}}$ is filter spatial size and $\:{\mathbf{m}}_{\mathbf{l}}\:$is the output as the feature map. Hence, the complexity of the method = $\:\varvec{X}$+ $\:\varvec{Y}$+ $\:\varvec{Z}$. The running time of the proposed method along with state-of-the-art methods is computed. This is shown in Fig. 13. It is observed that the proposed method has performed well when compared with other methods. 

## Conclusion

An unsupervised band selection strategy with a combination of dual partitioning and ranking-based methods is presented in this paper. The strategy helps to mine spectral features along with the spatial features from an image. The intrinsic information from the selected bands is elevated using hemispherical reflectance. Finally, extracted both spectral and spatial features are trained with CNN for image classification. The experiments were conducted on three benchmark datasets collected via AVIRIS Sensor namely Indian Pines, Salinas, and KSC. The proposed method is evaluated qualitatively and quantitatively. Qualitatively, the classification maps generated from the informative bands trained on CNN have effectively identified the classes on the dataset. An extensive quantitative analysis with accurate measures and MSD made the model robust and can be used to generalize the properties of the proposed model. The reported OA is 99.92% on Indian Pines, 99.94% on Salinas, and 97.23% on KSC datasets. Finally, it is noted that the model can effectively be used in mapping various crops like Corn, Soy, Fallow, and Lettuce. These accuracy values are in the range of 73.75-99.8%.

## Data Availability

Code and data related to this article can be obtained by emailing the corresponding author.

## References

[1] Dabbiru, L. et al. Fusion of synthetic aperture radar and hyperspectral imagery to detect impacts of oil spill in Gulf of Mexico. In:* 2015 IEEE International Geoscience and Remote Sensing Symposium (IGARSS)* 2015/7/ 2015, pp.1901–1904. IEEE.

[2] Navin, M. S. & Agilandeeswari, L. Multispectral and hyperspectral images based land use / land cover change prediction analysis: An extensive review. *Multimedia Tools Appl.* **79**, 29751–29774. 10.1007/s11042-020-09531-z (2020).

[3] Faltynkova, A., Johnsen, G. & Wagner, M. Hyperspectral imaging as an emerging tool to analyze microplastics: A systematic review and recommendations for future development. *Microplastics Nanoplastics*. **1**, 13–13. 10.1186/s43591-021-00014-y (2021).

[4] Prabhavathy, P., Tripathy, B. K. & Venkatesan, M. Unsupervised learning method for mineral identification from hyperspectral data. pp.148–160. (2021).

[5] Kumar, C. et al. Automated lithological mapping by integrating spectral enhancement techniques and machine learning algorithms using AVIRIS-NG hyperspectral data in Gold-bearing granite-greenstone rocks in Hutti, India. *Int. J. Appl. Earth Obs. Geoinf.* **86**, 102006–102006. 10.1016/j.jag.2019.102006 (2020).

[6] Skauli, T. et al. Hyperspectral Imaging Technology and Systems, Exemplified by Airborne Real-time Target Detection. In:* CLEO:2011-Laser Applications to Photonic Applications Washington, D.C.*, CMG5–CMG5. (2011).

[7] Vaddi, R. et al. Strategies for dimensionality reduction in hyperspectral remote sensing: A comprehensive overview. *Egypt. J. Remote Sens. Space Sci.* **27**, 82–92. 10.1016/j.ejrs.2024.01.005 (2024).

[8] Diwaker, M. K. C. et al. A comparative performance analysis of feature extraction techniques for hyperspectral image classification. *Int. J. Softw. Eng. Its Appl.* **10**, 179–188. 10.14257/ijseia.2016.10.12.15 (2016).

[9] Pan, C. et al. Adaptive edge preserving maps in markov random fields for hyperspectral image classification. *IEEE Trans. Geosci. Remote Sens.* **59**, 8568–8583. 10.1109/TGRS.2020.3035642 (2021).

[10] Prabukumar, M. & Sawant, S. Band clustering using expectation–maximization algorithm and weighted average fusion-based feature extraction for hyperspectral image classification. *J. Appl. Remote Sens.* **12**, 1–1. 10.1117/1.JRS.12.046015 (2018).

[11] Sawant, S. & Manoharan, P. Hyperspectral band selection based on metaheuristic optimization approach. *Infrared Phys. Technol.* **107**, 103295–103295. 10.1016/j.infrared.2020.103295 (2020).

[12] Uddin, M. P., Mamun, M. A. & Hossain, M. A. PCA-based feature reduction for hyperspectral remote sensing image classification. *IETE Tech. Rev.* **38**, 377–396. 10.1080/02564602.2020.1740615 (2021).

[13] Vaddi, R. & Manoharan, P. CNN based hyperspectral image classification using unsupervised band selection and structure-preserving spatial features. *Infrared Phys. Technol.* **110**, 103457–103457. 10.1016/j.infrared.2020.103457 (2020).

[14] Vaddi, R. & Manoharan, P. Hyperspectral image classification using CNN with spectral and spatial features integration. *Infrared Phys. Technol.* **107**, 103296–103296. 10.1016/j.infrared.2020.103296 (2020).

[15] Ye, Z. et al. Functional feature extraction for hyperspectral image classification with adaptive rational function approximation. *IEEE Trans. Geosci. Remote Sens.* **59**, 7680–7694. 10.1109/TGRS.2021.3052807 (2021).

[16] Zhang, F., Wang, Q. & Li, X. Hyperspectral image band selection via global optimal clustering. In:* 2017 IEEE International Geoscience and Remote Sensing Symposium (IGARSS) 2017/7// 2017*, pp.1–4. IEEE.

[17] Tejasree, G. & Loganathan, A. An extensive review of hyperspectral image classification and prediction: techniques and challenges.* Multimed. Tools Appl.* 2024. 10.1007/s11042-024-18562-9

[18] Tang, G., Jia, S. & Li, J. An enhanced density peak-based clustering approach for hyperspectral band selection. In:* 2015 IEEE International Geoscience and Remote Sensing Symposium (IGARSS) 2015/7// 2015*, pp.1116–1119. IEEE.

[19] Sun, W. et al. Correntropy-based sparse spectral clustering for hyperspectral band selection. *IEEE Geosci. Remote Sens. Lett.* **17**, 484–488. 10.1109/LGRS.2019.2924934 (2020).

[20] Datta, A., Ghosh, S. & Ghosh, A. Combination of clustering and ranking techniques for unsupervised band selection of hyperspectral images. *IEEE J. Sel. Top. Appl. Earth Observations Remote Sens.* **8**, 2814–2823. 10.1109/JSTARS.2015.2428276 (2015).

[21] Shrutika, S., Manoharan, P. & Samiappan, S. Ranking and grouping based feature selection for hyperspectral image classification. In:* Proceedings Asian Conference on Remote Sensing* pp.2305–2313. (2018) 2018.

[22] Bevilacqua, M. & Berthoumieu, Y. Multiple-feature kernel-based probabilistic clustering for unsupervised band selection. *IEEE Trans. Geosci. Remote Sens.* **57**, 6675–6689. 10.1109/TGRS.2019.2907924 (2019).

[23] Dou, Z. et al. Band selection of hyperspectral images using attention-based autoencoders. *IEEE Geosci. Remote Sens. Lett.* **18**, 147–151. 10.1109/LGRS.2020.2967815 (2021).

[24] Chang, C-I. & Liu, K-H. Progressive* Band Selection of Spectral Unmixing for Hyperspectral Im*agery. *IEEE Trans. Geosci. Remote Sens.* **52**, 2002–2017. 10.1109/TGRS.2013.2257604 (2014).

[25] Chein, I. C. et al. A joint band prioritization and band-decorrelation approach to band selection for hyperspectral image classification. *IEEE Trans. Geosci. Remote Sens.* **37**, 2631–2641. 10.1109/36.803411 (1999).

[26] Bajcsy, P. & Groves, P. Methodology for hyperspectral band selection. *Photogrammetric Eng. Remote Sens.* **70**, 793–802. 10.14358/PERS.70.7.793 (2004).

[27] Kim, J-H. et al. Covariance-based band selection and its application to near-real-time hyperspectral target detection. *Opt. Eng.* **56**, 053101–053101. 10.1117/1.OE.56.5.053101 (2017).

[28] Su, H. et al. Optimized hyperspectral band selection using particle swarm optimization. *IEEE J. Sel. Top. Appl. Earth Observations Remote Sens.* **7**, 2659–2670. 10.1109/JSTARS.2014.2312539 (2014).

[29] Pal, M. K. et al. Dimensionality reduction of hyperspectral data: band selection using curve fitting. In: (eds Larar AM, Chauhan P, Suzuki M, /4// 2016, pp.98801 W-98801 W. (2016).

[30] Jia, S. et al. A novel ranking-based clustering approach for hyperspectral band selection. *IEEE Trans. Geosci. Remote Sens.* **54**, 88–102. 10.1109/TGRS.2015.2450759 (2016).

[31] Wang, S. et al. Clustering by fast search and find of density peaks with data field. *Chin. J. Electron.* **25**, 397–402. 10.1049/cje.2016.05.001 (2016).

[32] Sawant, S. & Manoharan, P. A hybrid optimization approach for hyperspectral band selection based on wind driven optimization and modified cuckoo search optimization. *Multimedia Tools Appl.* **80**, 1725–1748. 10.1007/s11042-020-09705-9 (2021).

[33] Zhu, X., Li, N. & Pan, Y. Optimization performance comparison of three different group intelligence algorithms on a SVM for hyperspectral imagery classification. *Remote Sens.* **11**, 734–734. 10.3390/rs11060734 (2019).

[34] Sawant, S. S., Prabukumar, M., Samiappan, S. A. & Band Selection Method For Hyperspectral Image Classification Based On Cuckoo Search Algorithm With Correlation Based Initialization. In:* 2019 10th Workshop on Hyperspectral Imaging and Signal Processing: Evolution in Remote Sensing (WHISPERS) 2019/9// 2019*, pp.1–4. IEEE.

[35] Sawant, S., Prabukumar, M. & Samiappan, S. A modified Cuckoo Search algorithm based optimal band subset selection approach for hyperspectral image classification. *J. Spectr. Imaging*. 10.1255/jsi.2020.a6 (2020).

[36] Sawant, S., Prabukumar, M., & Samiappan, S. A. Band Selection Method For Hyperspectral Image Classification Based On Cuckoo Search Algorithm With Correlation Based Initialization. pp. 1–4. (2019).

[37] Sawant, S. S. & Manoharan, P. Unsupervised band selection based on weighted information entropy and 3D discrete cosine transform for hyperspectral image classification. *Int. J. Remote Sens.* **41**, 3948–3969. 10.1080/01431161.2019.1711242 (2020).

[38] Kalidindi, K. R., Gottumukkala, P. S. V. & Davuluri, R. Derivative-based band clustering and multi-agent PSO optimization for optimal band selection of hyper-spectral images. *J. Supercomputing*. **76**, 5873–5898. 10.1007/s11227-019-03058-3 (2020).

[39] Sawant, S. S. et al. Multi-objective multi-verse optimizer based unsupervised band selection for hyperspectral image classification. *Int. J. Remote Sens.* **43**, 3990–4024. 10.1080/01431161.2022.2105666 (2022).

[40] Manoharan, P. & Boggavarapu, P. K. L. N. Improved whale optimization based band selection for hyperspectral remote sensing image classification. *Infrared Phys. Technol.* **119**, 103948–103948. 10.1016/j.infrared.2021.103948 (2021).

[41] Phaneendra Kumar, B. L. N. & Manoharan, P. Whale optimization-based band selection technique for hyperspectral image classification. *Int. J. Remote Sens.* **42**, 5105–5143. 10.1080/01431161.2021.1906979 (2021).

[42] Paul, A. & Chaki, N. Band selection using spectral and spatial information in particle swarm optimization for hyperspectral image classification. *Soft. Comput.* **26**, 2819–2834. 10.1007/s00500-022-06821-6 (2022).

[43] Tschannerl, J. et al. MIMR-DGSA: Unsupervised hyperspectral band selection based on information theory and a modified discrete gravitational search algorithm. *Inform. Fusion*. **51**, 189–200. 10.1016/j.inffus.2019.02.005 (2019).

[44] Raju, A. et al. Airborne Hyperspectral Imagery for Band Selection Using Moth–Flame Metaheuristic Optimization. *J. Imaging 8*. 126. 10.3390/jimaging8050126 (2022). 10.3390/jimaging8050126 PMC9144346 35621891

[45] Patro, R. N., Subudhi, S. & Biswal, P. K. Spectral clustering and spatial Frobenius norm-based Jaya optimisation for BS of hyperspectral images. *IET Image Proc.* **13**, 307–315. 10.1049/iet-ipr.2018.5109 (2019).

[46] Vineet, K., Jurgen, H., Abdelhak, M. Z., Band selection for hyperspectral & images based on self-tuning spectral clustering. In:* European Signal Processing Conference*. pp.1–5. (2013).

[47] Barman, B. & Patra, S. Variable precision rough set based unsupervised band selection technique for hyperspectral image classification. *Knowl. Based Syst.* **193**, 105414–105414. 10.1016/j.knosys.2019.105414 (2020).

[48] Sawant, S. & Prabukumar, M. New framework for hyperspectral band selection using modified wind-driven optimization algorithm. *Int. J. Remote Sens.* 1–22. 10.1080/01431161.2019.1607609 (2019).

[49] Wei, Y. et al. Unsupervised hyperspectral band selection via multimodal evolutionary algorithm and subspace decomposition. *Sensors* **23**, 2129–2129. 10.3390/s23042129 (2023). 36850727 10.3390/s23042129 PMC9960512

[50] Habermann, M., Shiguemori, E., & Fremont, V. Unsupervised cluster-wise hyperspectral band selection for classification. *Remote Sens.* **14**10.3390/rs14215374 (2022).

[51] Manoharan, P. & Vaddi, R. Wavelet enabled ranking and clustering-based band selection and three-dimensional spatial feature extraction for hyperspectral remote sensing image classification. *J. Appl. Remote Sens.* **15**10.1117/1.JRS.15.044506 (2021).

[52] Mohan, A. & Venkatesan, M. HybridCNN based hyperspectral image classification using multiscale spatiospectral features. *Infrared Phys. Technol.* **108**, 103326–103326. 10.1016/j.infrared.2020.103326 (2020).

[53] Windrim, L. et al. Unsupervised Feature-Learning for Hyperspectral Data with Autoencoders. *Remote Sens.* **11**, 864–864. 10.3390/rs11070864 (2019).

[54] Vaddi, R. & Manoharan, P. Hyperspectral remote sensing image classification using combinatorial optimisation based un-supervised band selection and CNN. *IET Image Proc.* **14**, 3909–3919. 10.1049/iet-ipr.2020.0728 (2020).

[55] Zhang, X., Guo, Y. & Zhang, X. Hyperspectral image classification based on optimized convolutional neural networks with 3D stacked blocks. *Earth Sci. Inf.* **15**, 383–395. 10.1007/s12145-021-00731-1 (2022).

[56] Fırat, H., Asker, M. E. & Hanbay, D. Classification of hyperspectral remote sensing images using different dimension reduction methods with 3D/2D CNN. *Remote Sens. Applications: Soc. Environ.* **25**, 100694–100694. 10.1016/j.rsase.2022.100694 (2022).

[57] Jiang, Y., Li, Y. & Zhang, H. Hyperspectral Image Classification Based on 3-D Separable ResNet and Transfer Learning. *IEEE Geosci. Remote Sens. Lett.* **16**, 1949–1953. 10.1109/LGRS.2019.2913011 (2019).

[58] Ribalta Lorenzo, P. et al. Hyperspectral band selection using attention-based convolutional neural networks. *IEEE Access.* **8**, 42384–42403. 10.1109/ACCESS.2020.2977454 (2020).

[59] Santara, A. et al. BASS Net: Band-Adaptive Spectral-Spatial Feature Learning Neural Network for Hyperspectral Image Classification. *IEEE Trans. Geosci. Remote Sens.* **55**, 5293–5301. 10.1109/TGRS.2017.2705073 (2017).

[60] Cai, Y., Liu, X. & Cai, Z. BS-Nets: An End-to-End Framework for Band Selection of Hyperspectral Image. *IEEE Trans. Geosci. Remote Sens.* **58**, 1969–1984. 10.1109/TGRS.2019.2951433 (2020).

[61] Mou, L. et al. Deep reinforcement learning for band selection in hyperspectral image classification. *IEEE Trans. Geosci. Remote Sens.* **60**, 1–14. 10.1109/TGRS.2021.3067096 (2022).

[62] Agilandeeswari, L. et al. Crop classification for agricultural applications in hyperspectral remote sensing images. *Appl. Sci.* **12**, 1670–1670. 10.3390/app12031670 (2022).

[63] Boggavarapu, L. N. P. K. & Manoharan, P. A new framework for hyperspectral image classification using Gabor embedded patch based convolution neural network. *Infrared Phys. Technol.* **110**, 103455–103455. 10.1016/j.infrared.2020.103455 (2020).

[64] Kaul, A. & Raina, S. Support vector machine versus convolutional neural network for hyperspectral image classification: A systematic review. *Concurrency Computation: Pract. Experience*. **34**10.1002/cpe.6945 (2022).

[65] Eren, S. & Uysal, M. A comparative analysis of various activation functions and optimizers in a convolutional neural network for hyperspectral image classification. Multimedia Tools and Applications. ; 83: 1–32. DOI: (2023). 10.1007/s11042-023-17546-5

[66] Ladi, S. K. et al. A Novel Grey Wolf Optimisation based CNN Classifier for Hyperspectral Image classification. *Multimed Tools Appl.* **81**, 28207–28230. 10.1007/s11042-022-12628-2 (2022).

[67] Hitendra, T. et al. Kanthi. Band selection in Hyperspectral Images using information similarity ranking. doi: (2024). 10.1109/migars61408.2024.10544790

[68] Chang, L. & Guangping, W. Research on infrared hyperspectral band selection algorithm based on autoencoder. (2023). 10.1117/12.3007251

[69] Xiaorun, L., Yufei, L., Ziqiang, H. & Shuhan, C. An unsupervised band selection method via contrastive learning for hyperspectral images. Remote sensing, (2023). 10.3390/rs15235495

[70] Subhash, Chander, G., Chander, O. S., Goud., T. & Sarma., H. C., S., Bindu. Optimal band selection in hyperspectral images using improved K-means clustering with spectral similarity measures. (2023). 10.1109/iicaiet59451.2023.10291338

[71] Chander, O. S., Hitendra, G. & Sarma., C. S., Bindu. Improved K-means clustering algorithm for band selection in hyperspectral images. (2023). 10.1109/elexcom58812.2023.10370382

[72] Kinjal, D. Y., N., Trivedi. 8. A band selection method for crop classification based on spatial and spectral correlation using hyperspectral image. (2023). 10.1109/igarss52108.2023.10281569

[73] Wu, J. et al. A novel framework combining band selection algorithm and improved 3D prototypical network for tree species classification using airborne hyperspectral images. *Comput. Electron. Agric.* **219**, 108813. 10.1016/j.compag.2024.108813 (2024).

